# Proteomic analysis of human osteoarthritis synovial fluid

**DOI:** 10.1186/1559-0275-11-6

**Published:** 2014-02-17

**Authors:** Lavanya Balakrishnan, Raja Sekhar Nirujogi, Sartaj Ahmad, Mitali Bhattacharjee, Srikanth S Manda, Santosh Renuse, Dhanashree S Kelkar, Yashwanth Subbannayya, Rajesh Raju, Renu Goel, Joji Kurian Thomas, Navjyot Kaur, Mukesh Dhillon, Shantal Gupta Tankala, Ramesh Jois, Vivek Vasdev, YL Ramachandra, Nandini A Sahasrabuddhe, TS Keshava Prasad, Sujatha Mohan, Harsha Gowda, Subramanian Shankar, Akhilesh Pandey

**Affiliations:** 1 Institute of Bioinformatics, International Technology Park, Bangalore, Karnataka 560066, India; 2 Department of Biotechnology, Kuvempu University, Shankaraghatta, Shimoga, Karnataka 577451, India; 3 Centre for Excellence in Bioinformatics, School of Life Sciences, Pondicherry University, Pondicherry, Puducherry 605014, India; 4 Manipal University, Madhava Nagar, Manipal, Karnataka 576104, India; 5 Amrita School of Biotechnology, Amrita University, Kollam, Kerala 690525, India; 6 Rajiv Gandhi University of Health Sciences, Bangalore, Karnataka 560041, India; 7 Department of Internal Medicine, Armed Forces Medical College, Pune, Maharashtra 411040, India; 8 Department of Rheumatology, Fortis Hospitals, Bangalore, Karnataka 560076, India; 9 Department of Rheumatology, Command Airforce Hospital, Bangalore 560008, India; 10 Laboratory for Integrated Bioinformatics, RIKEN Center for Integrative Medical Sciences (IMS-RCAI), Yokohama Institute, Yokohama, Kanagawa 230-0045, Japan; 11 McKusick-Nathans Institute of Genetic Medicine, Johns Hopkins University, 733 N. Broadway, BRB 527, Baltimore, MD 21205, USA; 12 Department of Oncology, Johns Hopkins University School of Medicine, Baltimore, MD 21205, USA; 13 Department of Pathology, Johns Hopkins University School of Medicine, Baltimore MD 21205, USA; 14 Biological Chemistry, Johns Hopkins University School of Medicine, Baltimore, MD 21205, USA

**Keywords:** Body fluid, Cartilage, Joint destruction, Glycosylation

## Abstract

**Background:**

Osteoarthritis is a chronic musculoskeletal disorder characterized mainly by progressive degradation of the hyaline cartilage. Patients with osteoarthritis often postpone seeking medical help, which results in the diagnosis being made at an advanced stage of cartilage destruction. Sustained efforts are needed to identify specific markers that might help in early diagnosis, monitoring disease progression and in improving therapeutic outcomes. We employed a multipronged proteomic approach, which included multiple fractionation strategies followed by high resolution mass spectrometry analysis to explore the proteome of synovial fluid obtained from osteoarthritis patients. In addition to the total proteome, we also enriched glycoproteins from synovial fluid using lectin affinity chromatography.

**Results:**

We identified 677 proteins from synovial fluid of patients with osteoarthritis of which 545 proteins have not been previously reported. These novel proteins included ADAM-like decysin 1 (ADAMDEC1), alanyl (membrane) aminopeptidase (ANPEP), CD84, fibulin 1 (FBLN1), matrix remodelling associated 5 (MXRA5), secreted phosphoprotein 2 (SPP2) and spondin 2 (SPON2). We identified 300 proteins using lectin affinity chromatography, including the glycoproteins afamin (AFM), attractin (ATRN), fibrillin 1 (FBN1), transferrin (TF), tissue inhibitor of metalloproteinase 1 (TIMP1) and vasorin (VSN). Gene ontology analysis confirmed that a majority of the identified proteins were extracellular and are mostly involved in cell communication and signaling. We also confirmed the expression of ANPEP, dickkopf WNT signaling pathway inhibitor 3 (DKK3) and osteoglycin (OGN) by multiple reaction monitoring (MRM) analysis of osteoarthritis synovial fluid samples.

**Conclusions:**

We present an in-depth analysis of the synovial fluid proteome from patients with osteoarthritis. We believe that the catalog of proteins generated in this study will further enhance our knowledge regarding the pathophysiology of osteoarthritis and should assist in identifying better biomarkers for early diagnosis.

## Background

Osteoarthritis (OA) is a degenerative joint disorder characterized by articular cartilage damage, formation of osteophytes and subchondral bone cysts, thickened subchondral plate, inflammation and neovascularisation of synovial membrane [1]. OA is one of the leading causes of disability among the aging population. The two important risk factors for developing OA are obesity and age [2]. Despite the high prevalence of OA, its mechanism of pathogenesis still remains unclear [3]. The diagnosis of OA can be made based on structural abnormalities or symptoms resulting from these abnormalities. While OA is evident radiologically in most of the elderly population, only 10% are symptomatic and exhibit a measurable limitation of function. Further, radiographs may be normal in early disease owing to lack of sensitivity in visualizing minimal cartilage loss [4]. Thus, the diagnostic tools that are currently in use have their own limitations and provide an inaccurate assessment of disease progression [5]. Finally, the drugs currently used for the treatment of OA are aimed at reducing pain and do not possess any disease modifying activity [6].

Studying the synovial fluid proteome should yield a higher concentration of potential biomarkers than serum or plasma, as the synovial fluid is in direct physical contact with the synovium, ligament, meniscus, joint capsule and bone [7]. Alterations in the structure and metabolism of any of these tissues during disease should be reflected as alterations in the composition of the synovial fluid proteome. Therefore, the synovial fluid proteome has the potential to indicate the severity and progression of the disease [8]. Advances in proteomic technologies have facilitated extensive proteomic characterization of several body fluids [9-15]. A detailed molecular characterization of the synovial fluid could identify proteins associated with pathogenesis, which can be developed as markers for evaluation of the disease in early stages and its progression.

Yamagiwa *et al*. demonstrated a five-fold increase in the expression of 18 protein spots including haptoglobin among different synovial fluid samples from OA patients using 2-DE platform [16]. In another study, 135 proteins were identified from synovial fluid and 18 of them were shown to be differentially expressed in OA patients. Proteins identified to be elevated in OA included alpha 1- microglobulin, apolipoprotein E, complement component 3, haptoglobin, orosomucoid 1 and group specific component (vitamin D binding protein) [3]. A method of endogenous profiling of peptides from OA synovial fluid that resulted in identification of 40 proteins was described by Kamphorst *et al*. in 2007 [17]. In a recent study, abnormally high levels of complement components were shown in OA synovial fluid [18]. Sohn *et al*. identified 108 proteins from OA synovial fluid and found that only 36% of them were known to be in the plasma/serum [19]. Sixty six proteins, involved in acute phase response, complement and coagulation pathways were reported to be differentially expressed between healthy and OA synovial fluid in a recent study by Ritter *et al*[20]. A summary of previous proteomic studies on OA synovial fluid is provided in Table 1. Most of these investigations were carried out using low resolution mass spectrometers and with minimal fractionation of the samples, which limited the depth of coverage. In this study, we carried out a comprehensive cataloging of proteins from OA synovial fluid by including multiple fractionation methods followed by high resolution mass spectrometry analysis.

**Table 1:** A summary of proteomic studies published on healthy and osteoarthritis synovial fluid

	**Synovial fluid used (Healthy/Osteoarthritis)**	**Method**	**Mass spectrometer used**	**Number of proteins identified**	**Publication**
1	Healthy/Osteoarthritis	In-gel digestion	LCQ DECA XP	135	Gobezie, R *et al*., 2007 [3]
2	Osteoarthritis	Depletion of albumin & IgG, IEF, In-gel digestion	XCT Ultra Ion trap	108	Sohn, DH *et al*., 2012 [19]
3	Healthy/Osteoarthritis	2D-DIGE	-	66	Ritter, SY *et al.,* 2013 [20]
4	Healthy/Osteoarthritis	Ultrafiltration and solid phase extraction	LTQ XL-Orbitrap	40	Kamphorst, JJ *et al.*, 2007 [17]
5	Osteoarthritis	Depletion of albumin & IgG, 2-DE	-	18	Yamagiwa, H *et al.,* 2003 [16]
6	Healthy/Osteoarthritis	IEF, 2D-DIGE	-	12	Wang, Q *et al*., 2012 [18]
7	Osteoarthritis	Protein chip array	SELDI-TOF-MS	4	de Seny, D *et al*., 2011 [21]

## Results and discussion

### Identification of proteins from OA synovial fluid

**Figure 1:**
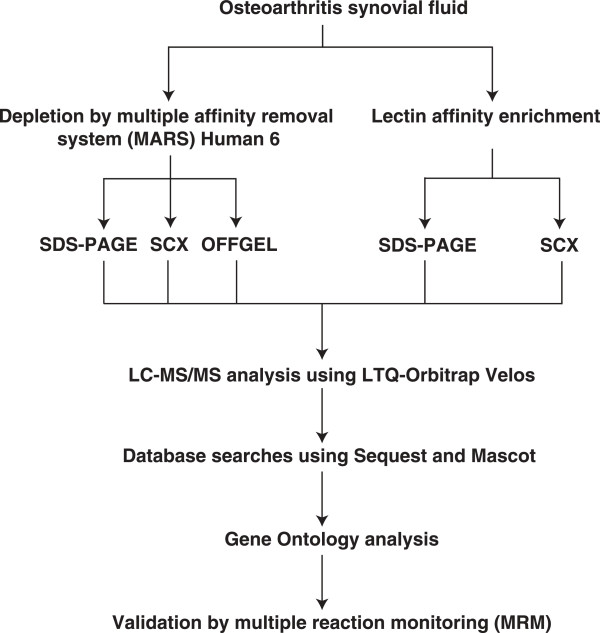
**Work flow illustrating the steps involved in the proteomic analysis of OA synovial fluid.** OA synovial fluid samples were pooled and subjected to depletion of abundant proteins by MARS-6 LC column and lectin affinity chromatography using three different lectins (Concanavalin A, wheat germ agglutinin and jacalin). The depleted fraction was then subjected to SDS-PAGE, SCX and OFFGEL fractionation. The lectin enriched fraction was subjected to SDS-PAGE analysis and SCX fractionation. All fractions were analyzed on LTQ-Orbitrap Velos mass spectrometer. Sequest and Mascot algorithms were used to perform database searches. Subsequently, gene ontology-based functional characterization of the identified synovial fluid proteins was carried out. Further, validation by MRM- based assays was carried out for three proteins identified from discovery studies.

Synovial fluid from five OA patients was pooled and the abundant proteins were depleted using Human MARS-6 column. The resulting sample was then subjected to multiple fractionation methods - SDS-PAGE at the protein level and SCX and OFFGEL at the peptide level - to reduce the complexity of the sample. In addition, lectin enrichment strategy was employed to enrich glycoproteins using a mixture of three different lectins - wheat germ agglutinin, concanavalin A and jacalin. These lectins have different binding specificities and thereby permit enrichment of a broader coverage of glycoproteins. The lectin enriched fractions were subjected to SDS-PAGE and SCX fractionation. All of these fractions were analyzed on a Fourier transform LTQ-Orbitrap Velos mass spectrometer. The workflow illustrating the steps involved in the proteomic analysis of OA synovial fluid is shown in Figure 1.

124,380 peptide spectrum matches generated from the mass spectrometric analysis of 112 fractions of depleted and lectin-enriched OA synovial fluid resulted in the identification of 5,544 peptides corresponding to 677 proteins. The number of proteins identified from the depleted and lectin-enriched fractions are summarized in Additional file 1A and 1B, respectively. Of the 300 lectin-enriched proteins identified, 171 proteins were already known to be glycosylated from the data available in Human Protein Reference Database (HPRD) [22,23]. The complete list of all proteins and peptides identified in our study are provided in Additional files 2 and 3, respectively. The relative abundance of the 25 most abundant proteins identified is provided in Additional file 4.

### Classification based on gene ontology (GO) annotation

**Figure 2:**
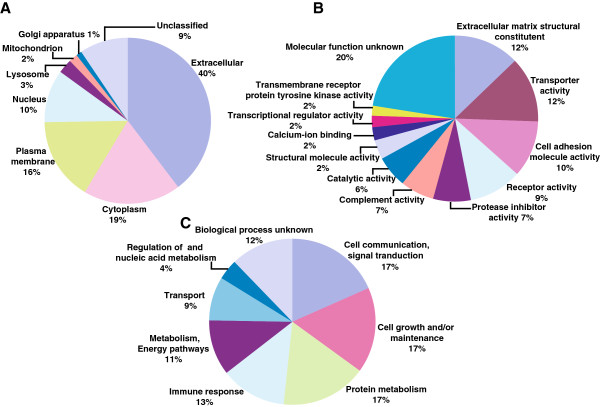
**Gene Ontology based classification of proteins identified from OA synovial fluid. (A)** Cellular component **(B)** Molecular function, and **(C)** Biological processes.

GO-based annotation was used to categorize the proteins based on their subcellular localization, molecular function and biological processes. Signal peptide and transmembrane domain analysis of the identified proteins was done by using the domains/motif information available in HPRD. Out of 677 proteins, 400 proteins were found to have a signal peptide, 113 have transmembrane domains and 77 proteins possessed both. Classification-based on the subcellular localization (Figure 2A) indicated that 40% of proteins were extracellular. Proteins were also localized to cytoplasm (19%), plasma membrane (16%) and nucleus (10%). Based on their molecular function (Figure 2B), proteins were classified as constituents of the extracellular matrix (12%) or those involved in transporter activity (12%), cell adhesion molecule activity (10%), protease inhibitor activity (7%) and complement activity (7%). Biological process-based (Figure 2C) categorization showed that a majority of them played a role in cell communication and signaling (17%), cell growth and/or maintenance (17%), protein metabolism (17%) and immune response (13%).

### Proteins previously reported in OA synovial fluid

Several proteins reported earlier in OA synovial fluid were identified in our study confirming the validity of the experimental approach employed by us. Collagen proteins provide the required strength and stiffness to the cartilage [24]. Several type I, III, V, and VI collagens (COL1A1, COL1A2, COL3A1, COL5A1, COL5A2 COL6A1 and COL6A3), aggrecan (ACAN), cartilage oligomeric protein (COMP), cartilage intermediate layer protein, matrix Gla protein, extracellular matrix protein 1, lumican and vitronectin identified in this study were already reported in OA synovial fluid [3,17]. ACAN is the major proteoglycan that confers load bearing properties to the cartilage [25]. The levels of COMP and ACAN were found to be significantly elevated in the serum and synovial fluid of OA patients [26,27] demonstrating its significance in OA pathogenesis. Xie *et al.* have shown an increased expression of fibronectin 1 (FN1) in the articular cartilage and synovial fluid of OA patients [28]. Matrix metalloproteinases (MMPs), MMP1 and MMP3 that were known to be involved in the degradation of extracellular matrix (ECM) of the cartilage were also identified in our study. Their levels were found to be higher in the synovial fluid of primary OA and joint knee injury patients [29]. The presence of several serine protease inhibitors (SERPINs), SERPINA1, SERPINA3, SERPINA6, SERPINC1, SERPINF1, SERPING1 that regulated the proteases involved in the degradation of ECM were also confirmed in our study [3,19]. Various complement components (C2 C3, C4A, C4B, C5, C7 and C9) that have been shown to contribute to the inflammation in OA joints were also identified in this study [18]. The levels of the primary lubricating macromolecule in synovial fluid, proteoglycan 4 (PRG4) has also been reported to be higher in the synovial fluid samples of patients in the advanced stage of OA [30].

### Proteins not reported in OA synovial fluid

**Figure 3:**
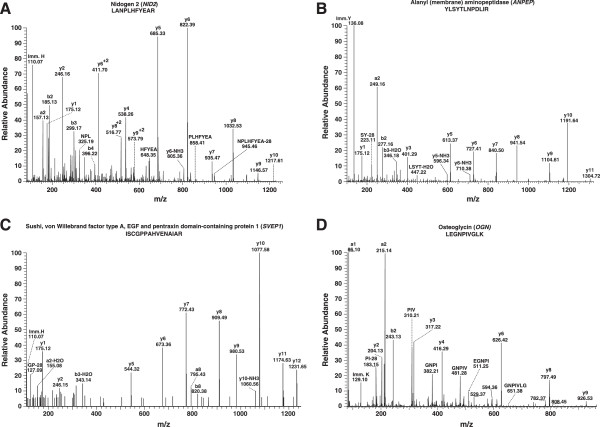
**Representative MS/MS spectra of peptides from novel proteins identified from OA synovial fluid. (A)** Nidogen 2 (*NID2*), **(B)** Alanyl (membrane) aminopeptidase (*ANPEP*), **(C)** Sushi, von Willebrand factor type A, EGF and pentraxin domain containing 1 (*SVEP1*) and **(D)** Osteoglycin (*OGN*).

Out of 677 proteins identified, 545 have not been reported earlier in OA synovial fluid. A partial list of novel proteins is provided in Table 2. Some of the novel molecules identified are discussed below. Representative MS/MS spectra of peptides identified from the proteins, Nidogen 2 (*NID2*), Alanyl (membrane) aminopeptidase (*ANPEP*), Sushi, von Willebrand factor type A, EGF and pentraxin domain containing 1 (*SVEP1*) and Osteoglycin (*OGN*) are shown in Figure 3.

**Table 2:** A partial list of proteins previously not reported in OA synovial fluid

	**Gene symbol**	**Protein**	**Subcellular localization**	**Molecule class**	**Molecular function**
1	*ADAMDEC1*	ADAM-like, decysin 1	Extracellular	Metalloprotease	Metallopeptidase activity
2	*ANPEP*	Alanyl (membrane) aminopeptidase	Plasma membrane, Extracellular	Metalloprotease	Metallopeptidase activity
3	*ASPN*	Asporin	Extracellular	Extracellular matrix protein	Extracellular matrix structural constituent
4	*CD84*	CD84 antigen (leukocyte antigen)	Plasma membrane	Immunoglobulin	Cell adhesion molecule activity
5	*COLEC10*	Collectin sub-family member 10 (C-type lectin)	Cytoplasm	Unclassified	Molecular function unknown
6	*DKK3*	Dickkopf WNT signaling pathway inhibitor 3	Extracellular, Cytoplasm	Ligand	Receptor and lipid binding
7	*MMRN2*	Multimerin 2	Extracellular	Extracellular matrix protein	Extracellular matrix structural constituent
8	*SPARCL1*	SPARC-like 1	Extracellular	Secreted polypeptide	Calcium ion binding
9	*THY1*	Thy-1 cell surface antigen	Plasma membrane	Integral membrane protein	Protein binding
10	*VSIG4*	V-set and immunoglobulin domain containing 4	Plasma membrane, Endosome	Complement receptor	Receptor activity

### Extracellular matrix proteins

Degradation of the articular cartilage is a hallmark of OA. Damage to the cartilage causes irreversible changes in the ECM that results in joint dysfunction [31]. Asporin (ASPN) is an ECM protein that belongs to the small leucine-rich proteoglycan family. Asporin was detected at higher levels in articular cartilage, subchondral bone and osteophytes of OA patients [32,33]. A recent study demonstrated that the expression of ASPN was highly regulated by the transcription factor, SP1 in the human articular chondrocytes [34]. Asporin has been shown to induce osteoblast-driven collagen mineralization [35]. Polymorphisms in the aspartic acid repeat of *ASPN* have been shown to be associated significantly with the susceptibility to OA [36]. Also, it has been shown to regulate chondrogenesis by inhibiting TGF-beta 1 mediated expression of genes, aggrecan (*ACAN*) and type II collagen (*COL2A1*) in the cartilage [36,37]. NID2 is a basement membrane protein that has been shown to interact with collagen type I, IV, laminin-1 and perlecan present in the ECM [38]. Kreugel J *et al.,* have shown that *NID2* expression was increased in late-stage OA cartilage in humans and established its role in cartilage regeneration [39].

### Proteolytic enzymes and protease inhibitors

Degradation of the ECM in OA synovial joint has been shown to be primarily catalyzed by the proteolytic enzymes. Alterations in the activities and expression levels of these enzymes and their associated inhibitors have been shown to disturb the balance between anabolism and catabolism in the affected joints [8]. Alanyl (membrane) aminopeptidase (ANPEP) is a membrane bound metalloprotease enzyme expressed on the surface of human normal and malignant myeloid cells, fibroblasts, hepatocytes and epithelial cells of the kidney and small intestine [40]. It has also been shown to be expressed by vascular endothelial cells and played a significant role in angiogenesis [41]. It has been suggested that simultaneous inhibition of ANPEP and dipeptidyl-peptidase 4 would provide an effective means of therapy against T-cell mediated disorders including autoimmune diseases, inflammation and allergy [42]. Recent studies have speculated its role in inflammatory monocyte trafficking [43]. ADAM-like, decysin 1 (ADAMDEC1) is a recently identified member of the disintegrin metalloproteinase family. It is located on the metalloprotease gene cluster on chromosome 8p12 comprising of other proteases, *ADAM7* and *ADAM28* and was shown to have arisen as a result of partial gene duplication of a gene located at this locus [44]. Abundant expression of ADAMDEC1 has been reported in monocytes-derived macrophages and in colon tissue [45].

Secreted phosphoprotein 2 (SPP2) is a 24 kDa secreted phosphoprotein initially cloned from bovine cortical bone. Northern blot analysis has shown *SPP2* expression in bone and liver. Its protein sequence was found to be related to the cystatin family of thiol protease inhibitors suggesting a role in the regulation of thiol proteases involved in bone turnover [46]. Studies have also suggested a role for SPP2 in the inhibition of calcification [47] and bone morphogenetic protein 2 (BMP-2) induced bone formation [48,49]. Serpin peptidase inhibitor, clade I (pancpin), member 2 (SERPINI2) belongs to the serine protease inhibitor superfamily. Though the other members of this superfamily have already been shown to be associated with OA, SERPINI2 has not been implicated in OA.

### Cell adhesion molecules

Cell-cell and cell-matrix interactions are mediated by cell adhesion molecules. These interactions are critical for the regulation of a plethora of biological processes including synovial inflammation and tissue remodelling [50]. Sushi, von Willebrand factor type A, EGF and pentraxin domain containing 1 (SVEP1) is a cell adhesion molecule, also known as selectin-like osteoblast derived protein. It was shown to be expressed in the skeletal cells of the bone and periosteum as well as by the stromal osteogenic cells [51]. The role of SVEP1 in mediating cell adhesion in an integrin α9β1dependent manner has been reported recently [52]. Osteomodulin (OMD) is a keratan sulfate proteoglycan that promotes cell binding mediated by integrin alphaV beta3 in bone [53]. Osteomodulin was detected in bovine mature osteoblasts and human odontoblasts suggesting its role in bone mineralization [54]. Its expression was found to increase the differentiation and maturation of osteoblasts [55]. Microarray analysis has revealed the association of Osteomodulin in osteoblast differentiation mediated by bone morphogenetic protein 2 [56].

### Growth factors and cytokines

Growth factors and cytokines are regulatory molecules that play a significant role in joint destruction and disease pathogenesis. Their levels are altered in case of joint injury or disease [8]. Osteoglycin (OGN), also known as mimecan or osteoinductive factor, belongs to the family of small leucine rich proteoglycans. Mice deficient in osteoglycin showed an increase in bone density [57]. In irradiated cultured osteoblasts, osteoglycin expression was elevated speculating its role in triggering the formation of bone along with other growth factors and matrix proteins [58]. Its expression was also increased in irradiated synovial membrane of rheumatoid arthritis patients [59]. Family with sequence similarity 3, member C (FAM3C) was characterized recently as a protein ubiquitously expressed in tissues with cytokine activity. It is also known as predicted-osteoblast protein, with no known function [60]. Polymorphisms in the *FAM3C* gene have been shown to be associated with bone mineral density and fore arm fracture [61,62].

### Glycoproteins in OA synovial fluid

Glycosylation of proteins is a biologically significant and complex post-translational modification associated with membrane and secreted proteins. Body fluids are rich in glycoproteins and characterizing the glycoproteome can increase the dynamic range of protein identification in synovial fluid [63]. We identified several glycoproteins in OA synovial fluid by lectin affinity enrichment. The list of all the proteins identified by lectin enrichment has been provided in Additional file 5. Afamin (AFM) is a vitamin E binding glycoprotein that belongs to the albumin gene family [64]. It was found to be secreted from differentiated osteoblasts and stimulated the migration of osteoblastic lineages through the activation of Akt signaling pathway [65]. Its presence in OA synovial fluid has been demonstrated by many proteomic studies [3,19]. Tissue inhibitor of metalloproteinases 1 (TIMP1) is a glycoprotein known to be involved in the degradation of extracellular matrix in the cartilage. TIMP1 levels have been demonstrated to be higher in the synovial fluid of OA knees with effusion [66]. C-type lectin domain family 3, member B (CLEC3B), also known as tetranectin is a plasminogen kringle-4 binding glycoprotein [67]. *CLEC3B* was involved in bone formation and was expressed at higher levels in the articular cartilage of OA patients [68]. Periostin (POSTN), also known as osteoblast-specific factor is a vitamin K-dependent protein. Expression of periostin was also detected in the periosteum and extracellular matrix of the cartilage and meniscus [69]. The association of periostin with bone mineral density and vertebral fracture risk has been recently illustrated by Xiao *et al.*[70].

### Validation by multiple reaction monitoring (MRM)

**Figure 4:**
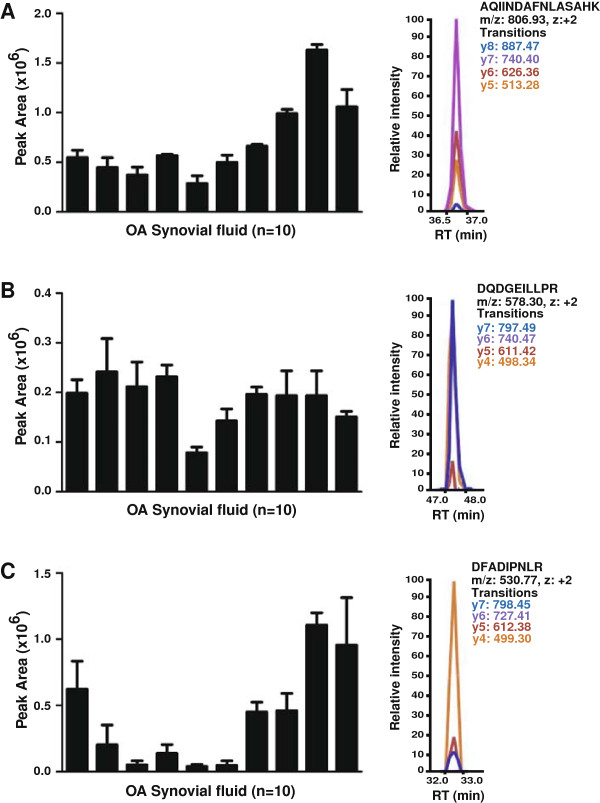
**Validation of proteins identified in OA synovial fluid by MRM analysis.** Bar graph representation of the peak area along with the MRM traces for the peptides validated by MRM. **(A)** Alanyl (membrane) aminopeptidase (*ANPEP*): AQIINDAFNLASAHK (z = +2, m/z = 806.93); **(B)** Dickkopf WNT signaling pathway inhibitor 3 (*DKK3*): DQDGEILLPR (z = +2, m/z = 578.30); **(C)** Osteoglycin (*OGN*): DFADIPNLR (z = +2, m/z = 530.77). (OA synovial fluid n = 10, RT: Retention time).

MRM analysis was employed to validate the expression of ANPEP, OGN and Dickkopf WNT signaling pathway inhibitor 3 (DKK3) in ten OA synovial fluid samples. These included the five samples that were used for the discovery phase LC-MS/MS analysis. ANPEP is a metalloprotease and OGN has growth factor activity and have been already described above. DKK3 is an antagonist of Wnt signaling pathway and its expression has been reported to be upregulated in the OA cartilage [71]. The proteotypic peptides selected for ANPEP were AQIINDAFNLASAHK (z = +2, m/z = 806.93) and YLSYTLNPDLIR (z = +2, 734.40). For OGN, the peptides targeted were DFADIPNLR (z = +2, m/z + 530.77) and LEGNPIVLGK (z = +2, m/z = 520.31). For DKK3, DQDGEILLPR (z = +2, m/z = 578.30) was targeted (Table 3). The MRM results from these experiments show that the proteins are easily detected in all individual OA synovial fluid samples in agreement with LC-MS/MS data obtained from the pooled samples. The bar graphs representing the peak areas from triplicate runs for each protein are shown in Figure 4.

**Table 3:** A list of peptides along with the transitions monitored for the proteins validated by MRM analysis

**Gene symbol**	**Protein name**	**Peptide sequence**	**Collision energy**	**Q1**	**Q3**	**Ion type**
*ANPEP*	Alanyl (membrane) aminopeptidase	AQIINDAFNLASAHK	30	806.93	887.47	y8
					740.40	y7
					626.36	y6
					513.28	y5
*ANPEP*	Alanyl (membrane) aminopeptidase	YLSYTLNPDLIR	27.4	734.40	840.49	y7
					727.41	y6
					613.37	y5
					516.31	y4
*DKK3*	Dickkopf WNT signaling pathway inhibitor 3	DQDGEILLPR	21.8	578.30	797.49	y7
					740.47	y6
					611.42	y5
					498.34	y4
*OGN*	Osteoglycin	DFADIPNLR	20.1	530.77	798.45	y7
					727.41	y6
					612.38	y5
					499.30	y4
*OGN*	Osteoglycin	LEGNPIVLGK	19.7	520.31	797.49	y8
					740.47	y7
					626.42	y6
					529.37	y5

### Data availability

The raw data obtained in this study were submitted to public data repositories, Human Proteinpedia (https://www.humanproteinpedia.org) and Tranche (https://www.proteomecommons.org/tranche/). Processed data and the database search results can be downloaded from Human Proteinpedia using HuPA_00698 code [72]. The following hash can be used to download the raw data from Tranche repository: jQquXSNp5ly3M7vOj66hnmxADXDp2DPU7BSyWzal5KdJPGKIxe6YFp2vVMPVDOaYCOD1DShgS4XN5gb87B4c/r9sE + sAAAAAAAA2CA==

## Conclusions

Using high resolution mass spectrometry, we have identified the largest number of OA synovial fluid proteins reported thus far. Multiple fractionation methodologies were employed to decrease the complexity of the sample and increase the depth of our analysis. We have identified 545 proteins that were not previously reported in OA synovial fluid. We also validated the expression of ANPEP, DKK3 and OGN in ten OA synovial fluid samples by MRM analysis. Some of these identified proteins can be further evaluated for their potential as specific targets or useful biomarkers for OA. These proteins could further enhance our knowledge and provide better insights regarding the underlying mechanism of OA pathogenesis perhaps leading to better therapeutic strategies.

## Methods

### Sample collection and processing

The samples were collected after obtaining informed consent of the patients and approval from the Institutional Ethical Committees of the Armed Forces Medical College, Pune, Fortis Hospitals, Bangalore and Command Air Force Hospital, Bangalore. Synovial fluid samples were collected from the affected joints of 10 OA patients, clinically diagnosed as per the criteria of American College of Rheumatology. These 10 OA patients included 7 females and 3 males with an average age of 65 years. Approximately 5 ml of synovial fluid was aspirated from each patient in heparin containing BD vacutainers (Becton, Dickinson and Company, New Jersey). The synovial fluid was then centrifuged at 1,500 g for 15 minutes and the supernatants were then filtered by using 0.22 μm filters (Catalog number: SLGV033RS Millipore, Massachusetts, USA) and stored at −80˚C until further processing. Twelve mg of protein isolated from five OA synovial fluid samples was pooled and depleted using Human 6-Multiple Affinity Removal LC Column (MARS-6) (Agilent Technologies, Santa Clara, USA) as per manufacturer’s instructions. The six most abundant proteins that are depleted using Human MARS-6 column are albumin, transferrin, haptoglobin, IgG, IgA, and alpha-1 antitrypsin. For each round of depletion, 1 mg protein was loaded onto the column and 12 such depletion runs were carried out. The elution of proteins was monitored at 280 nm. The depleted synovial fluid samples from each round were pooled and their protein concentration was estimated by Lowry’s method [73]. Protein from the depleted and pooled protein sample was subsequently fractionated by SDS-PAGE at protein level and by, strong cation exchange (SCX) chromatography and pI-based OFFGEL electrophoresis at peptide level.

### SDS-PAGE and in-gel digestion

300 μg of OA synovial fluid protein depleted of abundant proteins was resolved on a 10% SDS-PAGE (16X18cm). The gel was then stained using colloidal Coomassie blue. Twenty eight gel bands were excised and destained using 40 mM ammonium bicarbonate in 40% acetonitrile (ACN). In-gel digestion was carried out as described previously [74]. The sample was subjected to reduction using 5 mM DTT (60˚C for 45 minutes) followed by alkylation using 20 mM iodoacetamide (room temperature for 10 min in dark). Trypsin digestion was carried out at 37˚C for 12–16 hrs (Catalog number: V5111 Sequencing grade, Promega, Madison, WI, US). Peptides were extracted from gel pieces sequentially using 0.4% formic acid in 3% ACN twice, once using 0.4% formic acid in 50% ACN and once using 100% ACN. The extracted peptides were dried and stored at −80˚C until LC-MS/MS analysis.

### In-solution digestion

Five hundred μg of depleted synovial fluid protein was reconstituted in 40 mM ammonium bicarbonate. It was then reduced (5 mM DTT), alkylated (20 mM iodoacetamide) and digested overnight using trypsin as mentioned above.

### Strong cation exchange (SCX) chromatography

SCX was carried out as described earlier [75]. Briefly, 200 μg of digested peptide mixture was acidified using 1 M phosphoric acid and equilibrated with 10 mM potassium phosphate buffer containing 25% acetonitrile, pH 2.85 (solvent A) and fractionated using SCX on a Polysulfoethyl A column (PolyLC, Columbia, MD) (300 Å, 5 μm, 100 × 2.1 mm) using an Agilent 1200 HPLC system (Agilent Technologies, Santa Clara, USA) containing a binary pump, UV detector and a fraction collector. The peptides were eluted using a salt gradient (0 to 100%) between solvent A and solvent B (10 mM potassium phosphate buffer containing 25% acetonitrile, 350 mM KCl, pH 2.85). Twenty six fractions obtained from the fractionation were completely dried, reconstituted in 0.1% trifluoroacetic acid, and further desalted using stage-tips packed with C18 material [76]. Desalted fractions were dried in speedvac and reconstituted in 10 μl of 0.1% TFA prior to reversed-phase (RP) liquid chromatography based tandem mass spectrometry (LC-MS/MS) analysis.

### OFFGEL fractionation

Approximately 300 μg of in-solution digested depleted tryptic peptides was used for isoelectric point based fractionation using Agilent’s 3100 OFFGEL fractionator (Agilent Technologies, Santa Clara, USA). As per the manufacturer’s protocol, peptides were separated using pH 3–10 IPG strip. The peptides were focused for 50kVh with maximum current of 50 μA and maximum voltage set to 4000 V. Twelve fractions were collected after fractionation and then acidified using 1% TFA prior to sample cleaning using stage-tips [76].

### Lectin affinity enrichment

Approximately 10 mg of the total protein pooled from five OA samples was diluted in 10 mM phosphate buffer, pH 7.8. For glycoprotein enrichment, the samples were incubated with a mixture of three agarose conjugated lectins- concanavalin A (Con A), wheat germ agglutinin and jacalin (Vector labs, USA) for 12 h at 4˚C. The beads were then washed three times using wash buffer (10 mM phosphate buffer, pH 7.8) and the bound proteins were eluted using a mixture of carbohydrates (100 mM each of N-acetylglucosamine, melibiose and galactose). The eluate was dialyzed to remove free sugars and then concentrated using 3 kDa cut-off filters. The protein concentration was estimated by Lowry’s method. Two hundred and fifty μg of the enriched protein fraction was then resolved by SDS-PAGE. Twenty six gel bands were excised and subjected to in-gel trypsin digestion procedure as described in the previous section [74]. Two hundred and fifty μg of the enriched glycoprotein was also subjected to SCX fractionation as described earlier. Twenty fractions were collected and desalted using stage tips as mentioned above.

### LC-MS/MS analysis

Tandem mass spectrometric analysis of 112 fractions obtained from depleted total proteome and enriched glycoproteome was carried out using LTQ-Orbitrap Velos mass spectrometer (Thermo Scientific, Bremen, Germany) interfaced with Agilent 1200 (Agilent technologies, Santa Clara, CA, USA) nano liquid chromatography system. The LC system consisted of an enrichment column (3 cm × 75 μm, C18 material 5 μ particle size, 100 Å pore size) and an analytical column (10 cm × 75 μm, C18 material C18 material 5 μ particle size, 100 Å pore size) packed using pressure injection cell. Electrospray ionization source was fitted with an emitter tip 8 μm (New Objective, Woburn, MA) and maintained at 2000 V ion spray voltage. Peptide samples were loaded onto an enrichment column in 0.1% formic acid, 5% ACN for 15 min and peptide separation carried out using a linear gradient of 7-35% solvent B (90% ACN in 0.1% formic acid) for 60 minutes at a constant flow rate of 350 nl/min. Data was acquired using Xcalibur 2.1 (Thermo Scientific, Bremen, Germany). The MS spectra were acquired in a data-dependent manner in the m/z range of 350 to 1800 and survey scans were acquired in Orbitrap mass analyzer at a mass resolution of 60,000 at 400 m/z. The MS/MS data was acquired in Orbitrap mass analyzer at a resolution of 15,000 at 400 m/z by targeting top 20 most abundant precursor ions for fragmentation using higher energy collisional dissociation activation at 39% normalised collision energy. Single and unassigned charge state precursor ions were rejected. The dynamic exclusion option was enabled during data acquisition with exclusion duration of 60 seconds. Lock mass option was enabled for real time calibration using polycyclodimethylsiloxane (m/z, 445.12) ions [77].

### Data analysis

Mass spectrometry data was analyzed using multiple search engines to maximize the peptide identifications. Proteome Discoverer 1.3 (Thermo Scientific, Bremen, Germany) was used to carry out the peak list generation and database searches. Precursor mass range of 500 to 8,000 Da and signal to noise ratio of 1.5 were used as the criteria for generation of peak list files. NCBI Refseq 49 human protein database with known contaminants (32,967 entries) was used as a reference database. Sequest and Mascot algorithms were used to carry out database searches. The parameters used for database searches include trypsin as a protease with allowed one missed cleavage, carbamidomethyl cysteine as a fixed modification, and oxidation of methionine as a dynamic modification. Precursor ion mass error window of 20 ppm and fragment ion mass error window of 0.1 Da were allowed. The raw data obtained were searched against decoy database to calculate 1% false discovery rate cut-off score [78]. Spectra that matched to the contaminants and those that did not pass the 1% FDR threshold were not considered for analysis.

### Multiple reaction monitoring (MRM)

MRM assays were developed to validate the results of LC-MS/MS analysis for three target proteins. Skyline 2.1 was used for method development, data analysis and interpretation of the MRM results [79]. Proteotypic peptides for each protein were selected from the discovery LC-MS/MS experiments. Preference was given to proteotypic peptides with precursor charge +2 that did not contain cysteine or methionine. A minimum of four transitions were monitored for each peptide. Equal protein amounts from the individual OA synovial fluid samples were subjected to trypsin digestion as described earlier [10]. MRM of each sample was carried out in triplicates on TSQ Quantum Ultra (Thermo, San Jose, CA) interfaced with Easy nanoLC II (previously Proxeon, Thermo Scientific, Bremen, Germany). Peptides were enriched on a trap column (5 μm, 75 μm × 2 cm.) for 5 minutes with solvent A (5% ACN in 0.1% formic acid). The peptides were separated on analytical column (3 μm, 75 μm × 10 cm) with a linear gradient of 7-35% solvent B (95% ACN in 0.1% formic acid) for 60 min at a constant flow rate of 300 nl/min. Both columns were packed in-house using Magic C18 AQ (Michrom Bioresources). Spray voltage of 2.5 kV was applied and ion transfer tube was maintained at 275°C. MRM data was acquired with Q1 and Q3 set at resolution of 0.4 and 0.7 respectively. The collision energy for each transition was optimized in Skyline based on the preliminary results [80].

### Determination of the relative abundance of OA synovial fluid proteins

The relative abundance of proteins in OA synovial fluid was determined by calculating normalized spectral abundance factors (NSAF) for each protein identified in the study as previously described [81]. NSAF for a protein k was calculated by dividing the total number of peptide spectral matches (S) identified for protein k by protein length (L) and then divided by the sum of S/L ratio for all proteins.

### Bioinformatics analysis

Gene Ontology (GO) [82] analysis was done to identify the biological processes and the molecular function associated with the identified proteins. Subcellular localization, post-translational modifications, transmembrane domain and signal peptide information of the identified proteins were obtained from Human Protein Reference Database (HPRD) (http://www.hprd.org), which is a GO compliant database [22,23].

## Abbreviations

OA: Osteoarthritis; MARS: Multiple affinity removal system; GO: Gene ontology; ECM: Extracellular matrix; OGN: Osteoglycin; OMD: Osteomodulin; ASPN: Asporin.

## Competing interests

The authors declare that they have no competing interests.

## Authors’ contributions

AP, SS, SM and HG participated in the conception and study design. LB and MB collected the samples and performed the experiments. RSN, SA, SR and DSK carried out fractionation and mass spectrometry analysis of the samples. LB wrote the manuscript. LB and YS prepared the manuscript figures. LB, NS, SMS, JKT, RR, RG were involved in data analysis and interpretation. MD, NK, SGT, VV, NS edited the manuscript. RJ, YLR, TSKP, HG, SM and AP critically read and revised the manuscript. All authors read and approved the final manuscript.

## Supplementary Material

Additional file 1 **Summary of proteins identified from OA synovial fluid by using different fractionation methods. (A)** Venn diagram illustrating the number of proteins identified from depleted fraction using three different fractionation methods (SDS-PAGE, SCX, and OFFGEL). **(B)** Venn diagram illustrating the number of proteins identified from lectin enriched fraction subjected to SDS-PAGE and SCX fractionation. Click here for file

Additional file 2 **A list of proteins identified in this study.** This table lists all the synovial fluid proteins identified in the study along with the protein accession, gene symbol, protein description, coverage, unique peptides, number of peptide spectral matches (PSMs), amino acids, relative abundance, molecular weight and molecular function. Click here for file

Additional file 3 **A complete list of peptides identified in this study.** This table contains all the peptides identified in the study along with peptide sequence, protein description, gene symbol, protein group accession, XCorr, Ion Score, modifications, charge, m/z (Da), MH + (Da), Delta mass (ppm) and retention time (RT). Click here for file

Additional file 4 Relative abundance of twenty five most abundant proteins in OA synovial fluid. Click here for file

Additional file 5 **A list of proteins identified by lectin affinity enrichment.** This table includes a list of all the proteins identified by lectin affinity enrichment along with protein accession, gene symbol, protein description and post-translational modifications obtained from HPRD. Click here for file
